# A handy method to remove bacterial contamination from fungal cultures

**DOI:** 10.1371/journal.pone.0224635

**Published:** 2019-11-06

**Authors:** Xiao-Xiao Shi, Hai-Ping Qiu, Jiao-yu Wang, Zhen Zhang, Yan-Li Wang, Guo-Chang Sun

**Affiliations:** 1 State Key Laboratory for Managing Biotic and Chemical Threats to the Quality and Safety of Agro-products, Institute of Plant Protection Microbiology, Zhejiang Academy of Agricultural Sciences, Hangzhou, China; 2 College of Chemistry and Life Sciences, Zhejiang Normal University, Jinhua, China; Fujian Agriculture and Forestry University, CHINA

## Abstract

Contamination control and removal are very important technical aspects of microbiological research. Bacterial contamination is very common in fungal cultures. Currently, the commonly used approach for inhibiting bacteria is antibiotic treatment; however, there are drawbacks to using antibiotics, including incomplete removal, limited antibacterial spectra, tendency toward recontamination, effects to fungal strains, and potential risks to the environment. Therefore, in the present work, we developed a new method for bacterial removal from fungi cultured on solid medium, the Cabin-Sequestering (CS) method, based on the different culture characteristics between fungi and bacteria. First, 3–5 mm round or square holes (the “cabin”) are excavated on a solid medium plate. The fungal strain containing possible bacterial contamination is inoculated into the cabin. The cabin is then covered with a sterilized coverslip, followed by incubation at the appropriate temperature. After 7–10 days of culturing, fungal hyphae grow out along the edge of the coverslip; however, the contaminating bacteria cannot pass through the space formed between the medium and the coverslip and, thus, remain in the cabin. The newly grown fungal hyphae around the coverslip are re-inoculated into fresh culture plates, where they form bacteria-free fungal colonies. The CS method is easy handling, with a short experimental cycle and rare recontamination. When necessary, it can also be used in combination with antibiotics in bacterial removal operations.

## 1. Introduction

Contamination is a widely occurring phenomenon and a prominent problem in microbiological research and microbial production. Various kinds of microbial contamination can occur during the cultivation of fungal species, whither they are edible fungi, beneficial fungi and pathogenic fungi. The contaminated fungal strain must be purified [1, 2]. Microorganisms that contaminate fungal cultures mainly include bacteria and other fungi. Most of fungi such as molds usually grow rapidly on culture media, thus, the fungal strains contaminated by another fungus are easily found and also can be easily purified based on their colony morphology. However, contaminated bacteria often require a longer time, usually several generations of cultures, to form visible colonies on fungal cultures. Therefore, the bacterial contaminations is often more troublesome.

**Fig 1:**
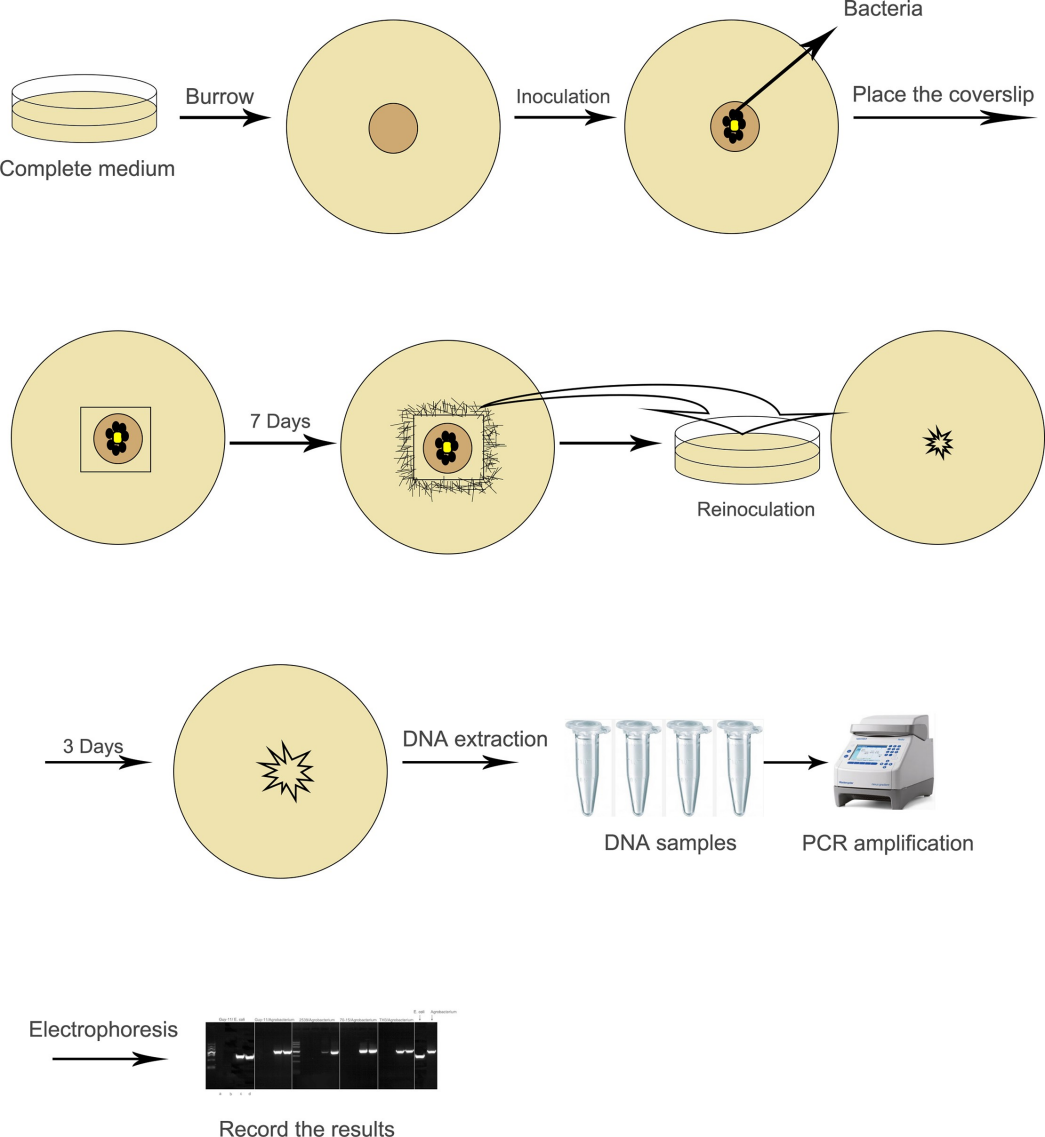
Fungal culture plates with bacterial contaminations. Some common bacterial contaminations during culturing *Magnaporthe oryzae* Guy-11 strains on complete medium plates.

On solid fungal culture media, bacterial contamination usually appears as white or light-yellow mucoid colonies. Based on the location of the bacterial contaminations, they are divided into surface-bacterial contaminations and endogenous-bacterial contaminations. Surface bacterial contaminations are often noticeable 1 to 2 days after inoculation [3, 4]. The contaminations caused by endogenous bacteria are not apparent at the beginning of inoculation. Along the rounds of subcultures, the bacteria accumulate gradually and become visible on the medium [3]. Fungi contaminated with bacteria tend to form colonies in different morphology from that of the normal ones; e.g., the colonies are maybe thinner or with lighter colors, fewer aerial hyphae, slower growth rates or reduced sporulation (Fig 1).

The common method for eliminating bacterial contamination is to supplement antibiotics into the medium. However, the antibiotics generally have their unique antibacterial spectra and no single antibiotic is effective against all bacteria. In practice, two or more antibiotics were often used simultaneously, however, even so, the outcomes are not always satisfied [5]. In addition, antibiotics are generally unstable and repeated use of a same antibiotic to control a bacterium tends to induce antibiotic resistance in the bacterium [6, 7]. Therefore, using antibiotics is not always a best approach to prevent bacterial contaminations [8]. Some reports suggest that the presence of organic materials in the medium is a reason for contamination [9], thus removing the organic materials from the medium is capable of reducing the chance of contamination. The method to inhibit bacteria by reducing organic materials in the medium is commonly used in plant tissue culture, but is not preferred for fungal culture, as fungi are not adapted for autotrophic reproduction like plants [10].

Cultured on solid media, fungi and bacteria exhibit their own characteristics distinctive to each other. The hyphae of most filamentous fungi spread rapidly on the surface of the medium or inside the medium and easily form visible colonies. The fungal species such as *Neurospora crassia*, *Penicillium* and *Magnaporthe oryzae*, can form colonies with a diameter of approximately 10 cm for only 4–10 days cultured on solid media [11, 12, 13]. Contrarily, bacteria expand in much slower speeds on solid media than that of fungi and they often only form colonies scattered or in lines along the inoculated sites. Some anaerobic bacteria and flagellated bacteria can also grow and expand inside solid media, however, much slower than fungi. Based on these differences cultural characteristics between fungi and bacteria, several special operations or utensils to remove bacteria were established. Machacek [14] introduced a method by placing the contaminated agar culture into molten cooled agar and meanwhile putting a coverslip on top to seal and prevent the bacterial contaminant, then harvesting the uncontaminated subsurface hypha. This method requires transfer at the right moment of agar cooling, is thus difficult to process with a uniform procedure on different culture media and also not suitable for thermolabile fungi. Ko et al. [15] described two methods for the removal of bacteria from contaminated cultures of *Phytophthora*, *Pythium* and *Botryosphaeria* by scraping the agar on the bottom of the contaminated cultures in both glass and plastic Petri dishes. For the cultures in plastic Petri dishes, a heated blade was used to pierce the bottom of the Petri dishes. For those in glass Petri dishes, the whole cultures were firstly overturned using a sterile scraper. These two methods require well-developed skills and easily introduce new contamination during operation. Cother and Priest [16] described a method by putting a small amount of contaminated agar culture in a Petri dish and covering a large piece of fresh agar, then placing a second small, fresh agar plug on top of the large agar. Once the mycelium has emerged in the second plug, transfer it onto a new agar plate. However, to handle a lager agar is also a difficult operation which easily brings new contaminations.

Thus, to develop new strategies to eliminate bacterial contamination is still required for the research and production of fungi. In the present work, we established a new simple, efficient and stable method, assigned as Cabin-Sequestering (CS), for removing bacterial contamination on solid fungal culture.

## 2. Materials and methods

### 2.1. Fungal strains and culture conditions

Guy-11, 2539, 70–15, and TH3 are commonly used *M*.*oryzae* strains. The strains were cultured in a complete medium (CM) according to routine procedures [13]. Long-term preservation was performed as the method described [17].

*Fusarium graminearum* was isolated from wheat Fusarium head blight samples in Ninghai, Zhejiang province in China (29.03 N, 121.42 E); *Colletotrichum gloeosporioides* was isolated from grape anthracnose samples in Yuyao, Zhejiang province in China (30.04 N, 121.16 E); *Rhizoctonia solani* was isolated from rice sheath blight samples in Taizhou, Zhejiang province in China (28.36 N, 121.36 E); *Botryosphaeria rhodina* was isolated from grapevine cankers samples in Yuyao, Zhejiang province in China. These strains were cultured and preserved in our laboratory using traditional methods, and the media used were either CM or potato dextrose agar (PDA).

### 2.2. Operational procedures

**Fig 2:**
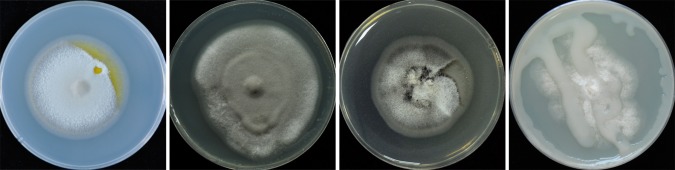
Experimental procedure of CS method.

The CS method consists of the following steps (Fig 2), and all steps are performed on a clean bench:

1. The appropriate solid media are prepared, autoclaved, and plated (with or without antibiotics as needed), and the cover slips and inoculation needles should also be sterilized.
2. A sterile inoculation needle or hole puncher is used to introduce a (or more) square or circular hole (the “cabin”) of approximately 3–5 mm in the appropriate solid medium;
3. A fungal hypha or colony containing potential bacterial contamination is picked with a sterile inoculation needle and inoculated into the cabin.
4. A sterilized coverslip is picked up using sterile forceps and carefully placed over the inoculated cabin. The coverslip is then gently pressed to ensure close contact with the medium with no air bubbles in between.
5. The culture plate is then covered and incubated for 7–10 days at the temperature appropriate for fungal growth; the number of days of growth can be reduced or increased depending on the growth rate of the fungal strain.
6. The development of fungal strains is checked regularly. Once the fungal hyphae grow out of the edge of the coverslip, they are picked and re-inoculated onto new culture plates. The new plates are then incubated under suitable conditions.
7. The new formed fungal colonies are checked for whether the bacterial contamination is removed based on their morphology and confirmed by polymerase chain reaction (PCR) detecting the bacterial DNA.
8. If necessary (the fungal strain still contains bacterial contamination), the above steps are repeated 2–3 times.

To evaluate effectiveness of the CS method, we selected four *M*. *oryzae* strains (Guy-11, 2539, 70–15, TH3) and four important plant pathogenic fungi as the tested fungal strains. Appropriate amounts of *E*. *coli* or *Agrobacterium tumefaciens* were manually added into the strains. To compare the effectiveness of the CS method with that of antibiotic treatment, we set four treatment groups: CS without antibiotics; CS with antibiotics; antibiotic treatment alone; and sub-culturing without CS or antibiotics. For antibiotic treatments, a combination of streptomycin sulfate (0.20 mg/ml) and potassium penicillin (0.20 mg/ml) were used. After the above treatments, each fungal sample was transferred into 30 progeny cultures. The capability of bacterial removal of the treatments was evaluated based on the morphology of the progeny colonies and the PCR test. The efficiency (E) of the bacterial removal was calculated basing on the PCR results as the equation E = N/T×100% (N represents the number of the progeny colonies without bacteria, and T represents the total progeny colonies). All of the experiments were repeated 3 times.

### 2.3. DNA extraction and PCR analysis

To test whether the bacteria were truly removed, PCR was used to detect the bacterial DNA fragments in the newly formed progeny fungal colonies. Fungal hyphae cultured in liquid CM for 3 days were harvested for genomic DNA extraction using the cetyltrimethylammonium bromide (CTAB) method [13]. The primers pairs EcChk1 (5’- CCGTTCCGCATTCGTGTTATTGAG -3’) / EcChk2 (5’- ATCGGCACCATCGCATCTTTCTTG -3’) and AtChk1 (5’-GGTGCTGGCAAAACCACCGCACTC -3’) / AtChk2 (5’- TCACCAATTGCTCGATGGCTTCTC -3’) were designed basing on the genome sequences of *E*. *coli* and *A*. *tumefaciens* respectively. The DNAs from pure bacteria cultures were used as the positive controls, and the sterilized water (dd H_2_O) as the negative control. PCR and electrophoresis were performed as standard procedures. More than 30 progeny colonies for each treatment were detected and the experiments were repeated three times. The bacterial removal efficiencies of the treatments were calculated by the percentages of colonies without bacterial DNA, and statistically compared.

## 3. Results and analysis

### 3.1. Assessment of the effectiveness of CS methods

**Fig 3:**
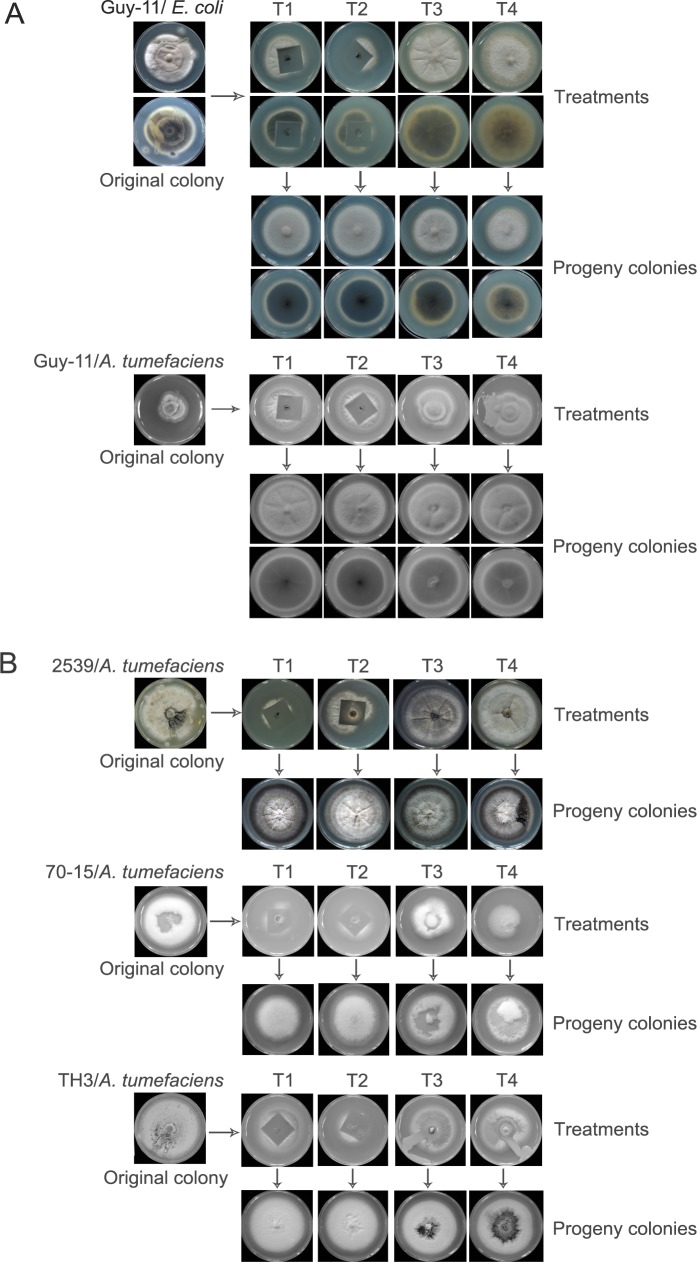
Comparison the treatments basing on CS method and antibiotic inhibitions. (A) *M*. *oryzae* Guy-11 strain mixed with *E*. *coli* or *A*. *tumefaciens* were treated using 4 different methods; (B) The treatments with *M*. *oryzae* 2539, 70–15, and TH3 strains mixed with *A*. *tumefaciens*. T1, CS treatment without antibiotics; T2, CS treatment in combination with antibiotics; T3, antibiotics only; T4, subculture on antibiotic-free media.

To evaluate the CS method, we set up 4 treatments (see the Methods section) to compare the CS method with the antibiotic method. The efficiencies of the treatments were assessed based on morphology of the progeny fungal colonies. In CS treatments, *M*. *oryzae* hyphae mixed with *E*. *coli* or *A*. *tumefaciens* were inoculated the cabins. The *M*. *oryzae* hyphae can penetrate into the surrounding medium and bypass the coverslip to grow and form new colonies around it. In contrast, the bacteria are restricted by the solid medium and the coverslip, and, thus, they are retained in the cabins under the coverslip. The fungal hyphae around the coverslip are re-inoculated onto new CM plates. An entire round of the CS method takes approximately 10 days. Using the CS method, the contaminating bacteria were largely removed, and the progeny fungal colonies exhibited a much better morphology than the original contaminated strains (Fig 3A). The CS treatments with and without antibiotics gave equivalent results, without obvious difference. However, only a few bacteria-free progeny fungal cultures were produced in the group with only antibiotic treatment after the two rounds of sub-culturing. In the blank control treatment, in which no antibiotics were used and only hyphae that appeared to be free of bacterial contamination were picked, most of the progeny cultures were still contaminated after second round of subculturing. The treatments carried out with the four *M*. *oryzae* strains (Guy-11, 2539, 70–15, and TH3) gave the similar results (Fig 3B), indicating that the CS method is superior to antibiotics-based approaches for removing bacterial contaminations. Interestingly, we found that the morphological characteristics of the progeny fungal colonies after CS treatments were significantly improved, and, in some cases, they even appeared healthier than their uncontaminated parental strains (S1 Fig). These facts suggest that the CS approach may be also capable of improving the development characteristics of the fungal strains.

### 3.2. Molecular confirmation of bacterial removal

**Fig 4:**
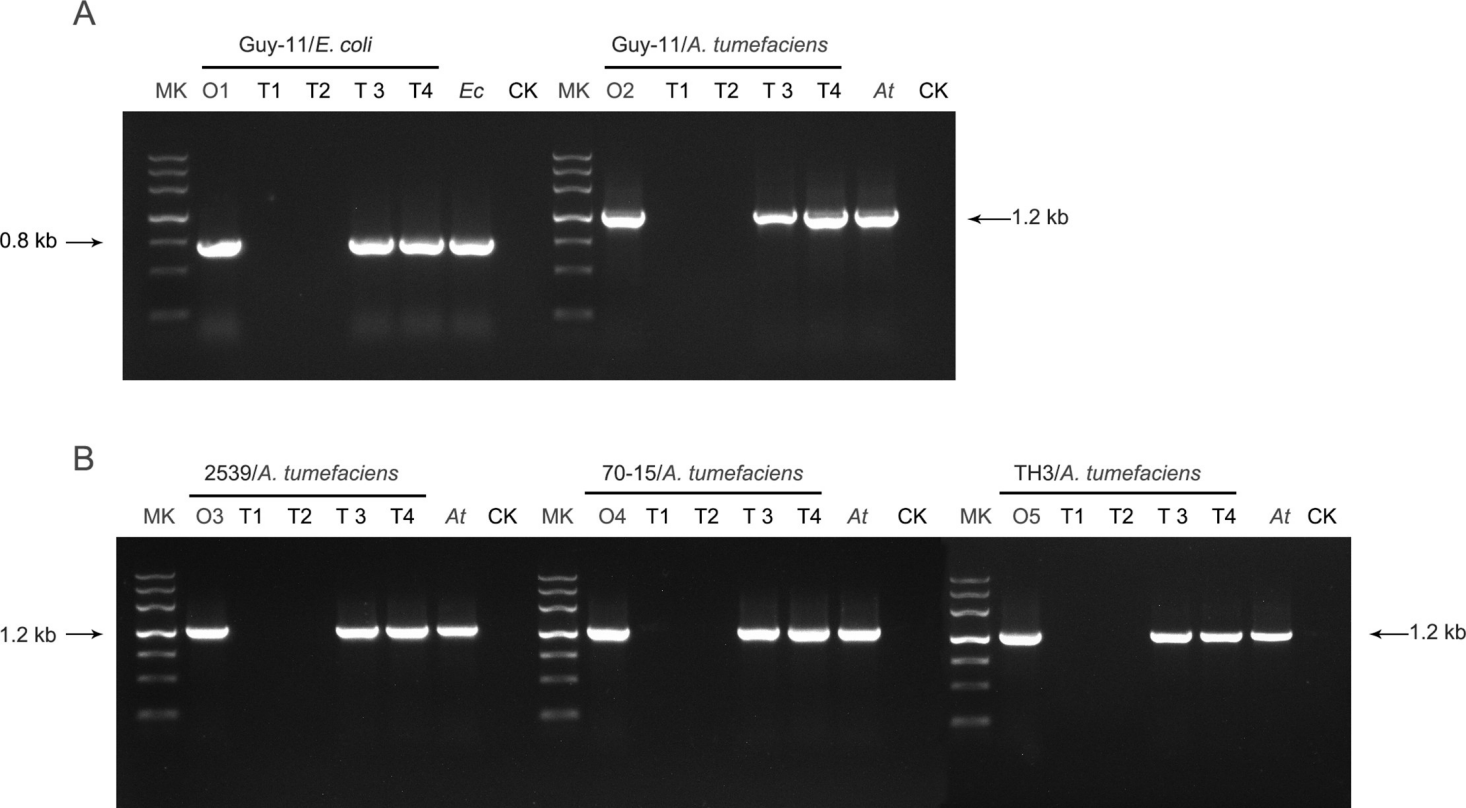
PCR detection of bacterial DNA in progeny fungal colonies after the treatments. PCR were performed using *E*. *coli* or *A*. *tumefaciens* specific primers and fungal DNA samples from *M*. *oryzae* Guy-11 strains contaminated with *E*. *coli* or *A*. *tumefaciens* (A) and *M*. *oryzae* 2539, 70–15, and TH3 strains contaminated with A. tumefaciens (B), as templates. O1-O5, the original contaminated strains before treatments; T1, CS treatment without antibiotics; T2, CS treatment in combination with antibiotics; T3, antibiotics only; T4, subculture on antibiotic-free media. The pure *E*. *coli* cultures (Ec) and *A*. *tumefaciens* (At) cultures were used as the positive controls, and the sterilized water (CK) as the negative controls. Mk, DNA Marker III (Tiangen Biotech Co.,Ltd, Beijing, China), a DNA ladder with bands in 4500, 3000, 2000, 1200, 800, 500 and 200 bp.

PCR amplifications were performed to detect whether the bacterial DNAs were still present in fungal colonies after the removal treatments (Fig 4). The results showed that for the CS method with or without antibiotics, the bacterial removal efficiencies in the progeny fungal strains were 95±3% and 97±3%, respectively; however, the difference was not statistically significant at a P = 0.05 level. With antibiotic treatment, the efficiency of bacterial removal in the progeny cultures (produced after the second round of subculturing) was only 15%. The treatment without antibiotics and relying only on subculturing of seemingly contamination-free colonies resulted in removal efficiencies less than 5% after the second rounds of subculturing. The PCR amplification test results were consistent with the morphological observations of the progeny colonies. The data indicates that the CS method is a better approach for clearing bacterial contamination relative to antibiotic inhibition.

### 3.3. Application of CS method in other fungal strains

**Fig 5:**
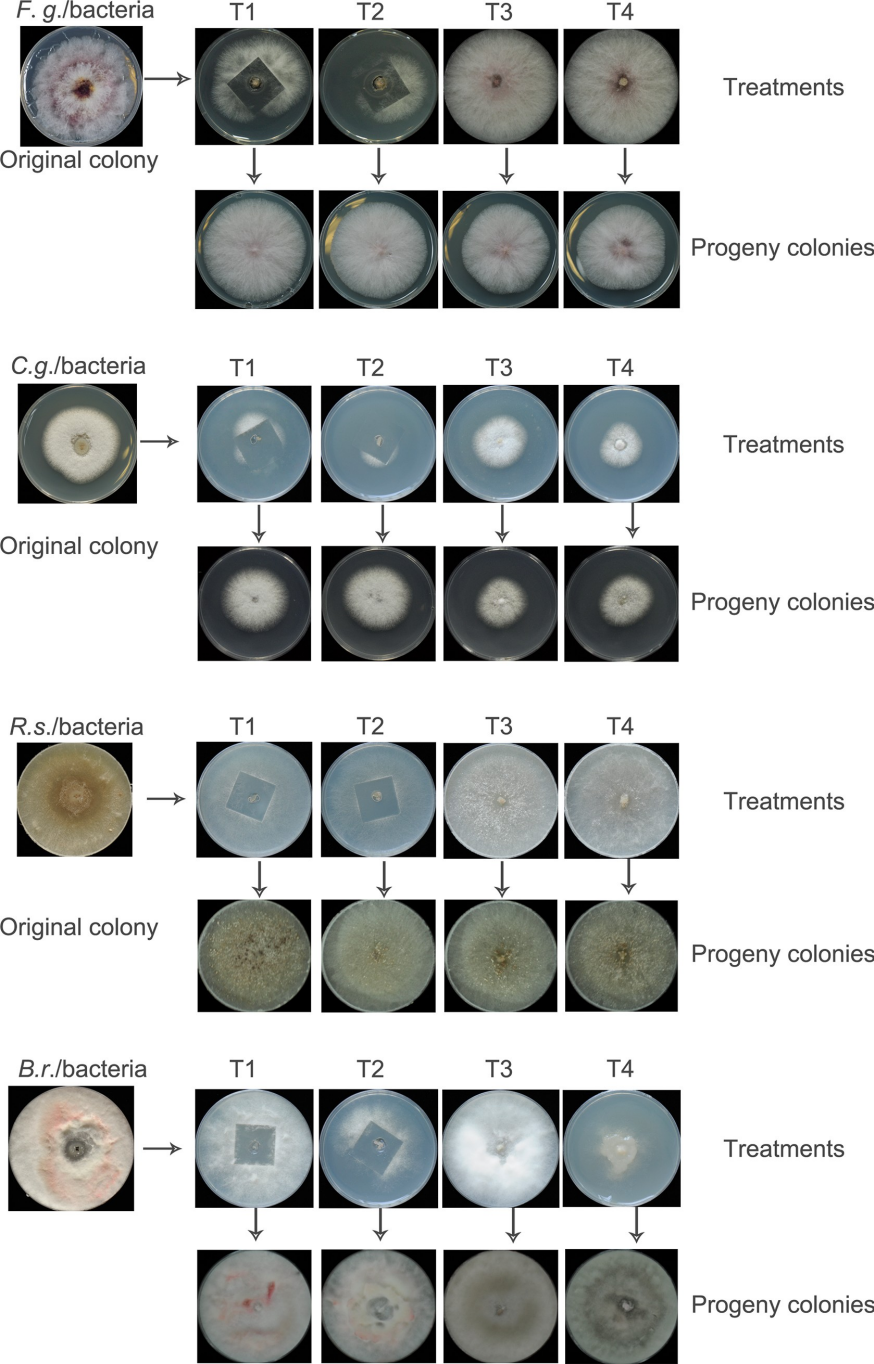
CS treatments and antibiotics inhibition for removing bacteria from different fungal strains. The naturally bacteria contaminated fungal strains of *F*. *graminearum*, *C*. *gloeosporioides*, *R*. *solani*, and *B*. *rhodina* were treated using the 4 different procedures; T1, CS treatment without antibiotics; T2, CS treatment in combination with antibiotics; T3, antibiotics only; T4, subculture on antibiotic-free media.

To further verify the efficacy of the CS method for other fungal species, the naturally contaminated strains of four major plant pathogenic fungi, *F*. *graminearum*, *C*. *gloeosporioides*, *R*. *solani*, and *B*. *rhodina* were processed with the same four treatments. The results indicated that, similar to the results with *M*. *oryzae* strains, the 4 fungal species were effectively decontaminated using the CS method with or without antibiotics. The status of the fungal colonies was also notably improved (Fig 5).

## 4. Discussion

Fungi cover divers of economically important microorganisms [18, 19]. Bacterial contamination is a broad problem that must be overcome in fungal production and research. In this paper, we have established the CS method for removing bacterial contamination from fungal colonies. From the results of the four sets of experiments, the effectiveness of the CS method appears to be superior to that of the antibiotic-based approaches in all of the tested fungal strains. Compared with the methods previously described [14, 15, 16], the CS procedure avoid transferring the medium at such a strict time like the first method [14] and doesn’t need high-developed skills as the second method [15]. In addition, the CS method can perform on different culture media and suit to different types of fungi. Furthermore, in CS method, we do not need handle a lager agar like that in the second and the third method, thus rarely bring new contaminations during operation. The CS method is easy to repeat and economical by not requiring antibiotics and it doesn’t take long to complete the entire process.

In our experiments, the bacteria were intentionally added to the fungal strains, in a much higher quantities than that of the majority of natural bacterial contaminations. Therefore, the efficiency of CS method was better in most of the actual practice in our lab. We have used the CS method to remove at least 300 bacterial contaminations from different fungal species involving different bacterial strains. In some cases, even bacteria that are extremely difficult to be distinguished from fungal colonies and hard to remove with conventional antibiotic treatments were successfully cleared by using CS method. Most of the bacteria in contaminated fungi can be removed by just one cycle of the CS procedure. In some minor cases, we require a second or third CS round to achieve the desired decontamination results. The morphological characteristics of fungal strains can be restored back to normal after removal of the bacteria, and some fungal strains even appeared healthier than the original uncontaminated strains.

A troublesome problem in culturing plant pathogenic fungi, such as *M*. *oryzae*, is strain degeneration over culture time and during subculturing, leading to weakened viability, reduced sporulation or decreased pathogenicity [20]. The reasons for these problems are not fully clear yet, but they are thought to be related to molecular processes, such as gene expression and posttranslational modification in the strains. Therefore, we have to preserve the original fungal strain over long periods, minimize the numbers of subcultures or frequently rejuvenating the fungal strains. Routinely, the fungal strain rejuvenation is performed by re-inoculating the strains into their hosts, which is a long and complicated process. We have found that after CS treatment, aside from the removal of the contaminating bacteria, the fungal characteristics, including sporulation, melanin production and pathogenicity, were also improved, while antibiotic treatment rarely produced such effects. The reason for this phenomenon may be that the fungus growing in the cabins has to break through the restrictions imposed by the coverslip and culture medium to grow out, which likely represents an automatic screen for fungal cells in better health and higher vitality. That is to say, the CS treatment not only removes the bacteria contamination, it also simultaneously allows the fungus to undergo an auto-activation or rejuvenation, and undoubtedly, the CS method is much easier than re-inoculating the fungal strains back into its host and subsequent re-isolating them.

Because they have different respiration patterns and motility, the bacteria vary in abilities to grow and migrate on or inside solid media. Theoretically, anaerobic and flagellated bacteria are better at growing and expanding inside media. *E*. *coli* is an anaerobe, whereas *Agrobacterium* is aerobic, and both species have flagella. In our experiments, using the CS method, *E*. *coli* and *A*. *tumefaciens* were both efficiently removed from different fungal strains, indicating that, the motility of the bacteria likely does not change the effectiveness of the CS method. This point was also confirmed by our practices using the CS method to treat various bacterial contaminations in multiple fungal strains. However, it cannot be completely ruled out that some bacteria may be able to go through the culture medium and grow out of the small cabin together with the fungal hyphae. Therefore, we prefer to pick the newly generated hyphae around the coverslip as soon as possible and to only pick the hyphal tips. Furthermore, for bacteria that are extremely difficult to remove, the CS procedure can be repeated multiple times or used in conjunction with antibiotics to achieve the optimal results. In summary, we established an easy-to-handle and highly effective bacterial contamination removal approach, the CS method, for fungal culture.

## Supporting information

S1 Fig Colonial morphology and conidiation of a fungal strain before and after CS. An *Magnaporthe oryzae* Guy-11 strain degenerated during repeat subculturing were treated by CS. After CS treatment, the colonial morphology and conidiation of the strains were improved were improved obviously.
(TIF)
