# Correction: A handy method to remove bacterial contamination from fungal cultures

**DOI:** 10.1371/journal.pone.0228293

**Published:** 2020-01-21

**Authors:** Xiao-Xiao Shi, Hai-Ping Qiu, Jiao-yu Wang, Zhen Zhang, Yan-Li Wang, Guo-Chang Sun

The images for Figs 1 and 2 are incorrectly switched. The image that appears as Fig 1 should be Fig 2, and the image that appears as Fig 2 should be Fig 1. The figure captions appear in the correct order.

**Fig 1:**
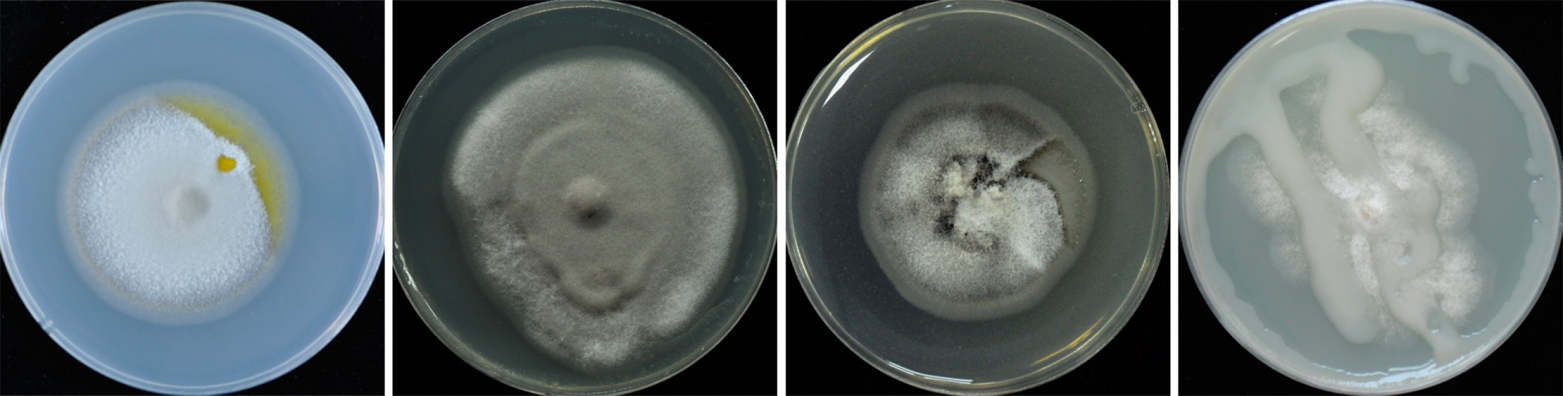
Fungal culture plates with bacterial contaminations. Some common bacterial contaminations during culturing *Magnaporthe oryzae* Guy-11 strains on complete medium plates.

**Fig 2:**
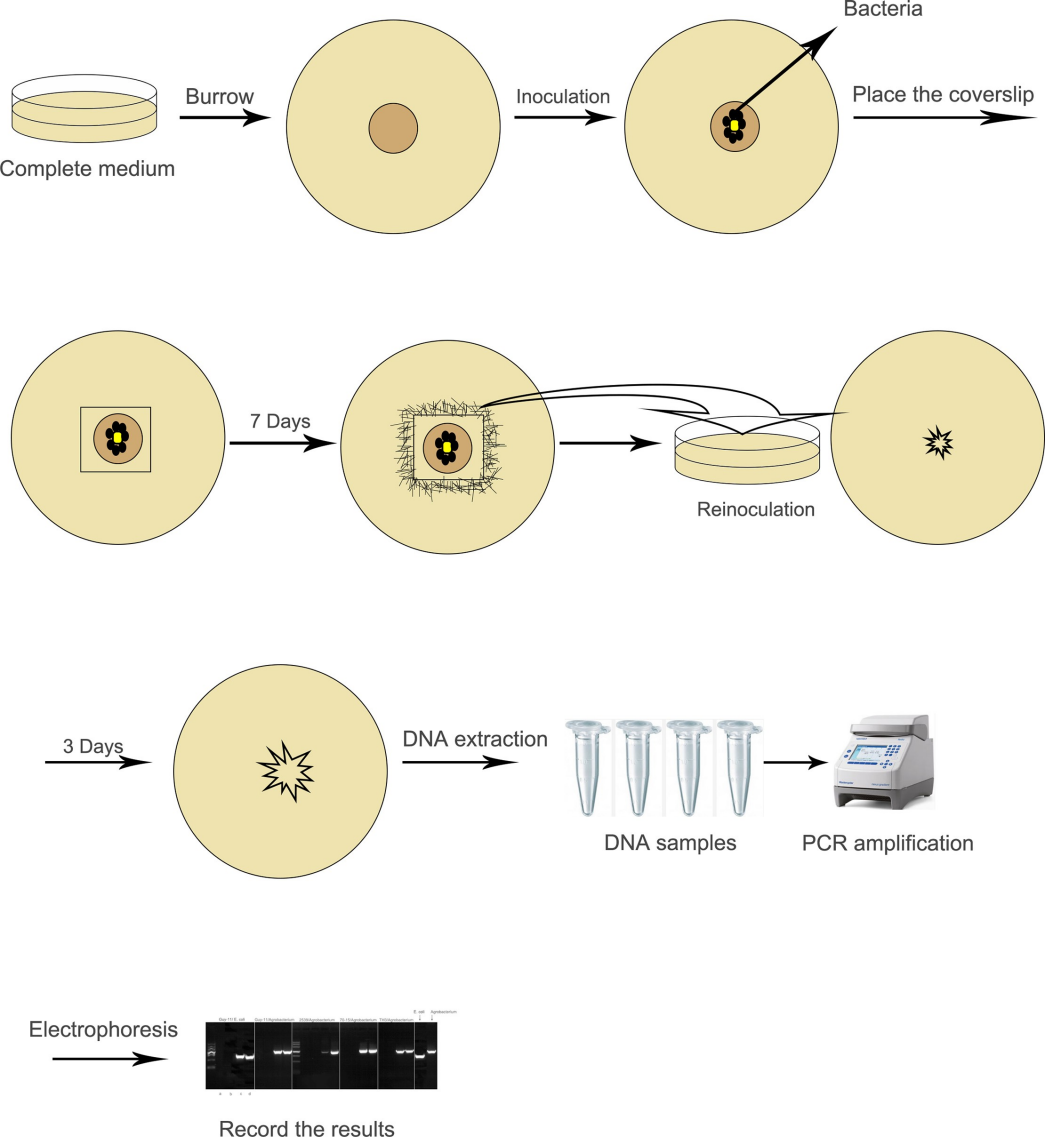
Experimental procedure of CS method.
