# Supplementary figures and images for: A handy method to remove bacterial contamination from fungal cultures
Source: PLoS One. 2019 Nov 6;14(11):e0224635. doi: 10.1371/journal.pone.0224635 (PMC6834272; doi:10.1371/journal.pone.0224635)

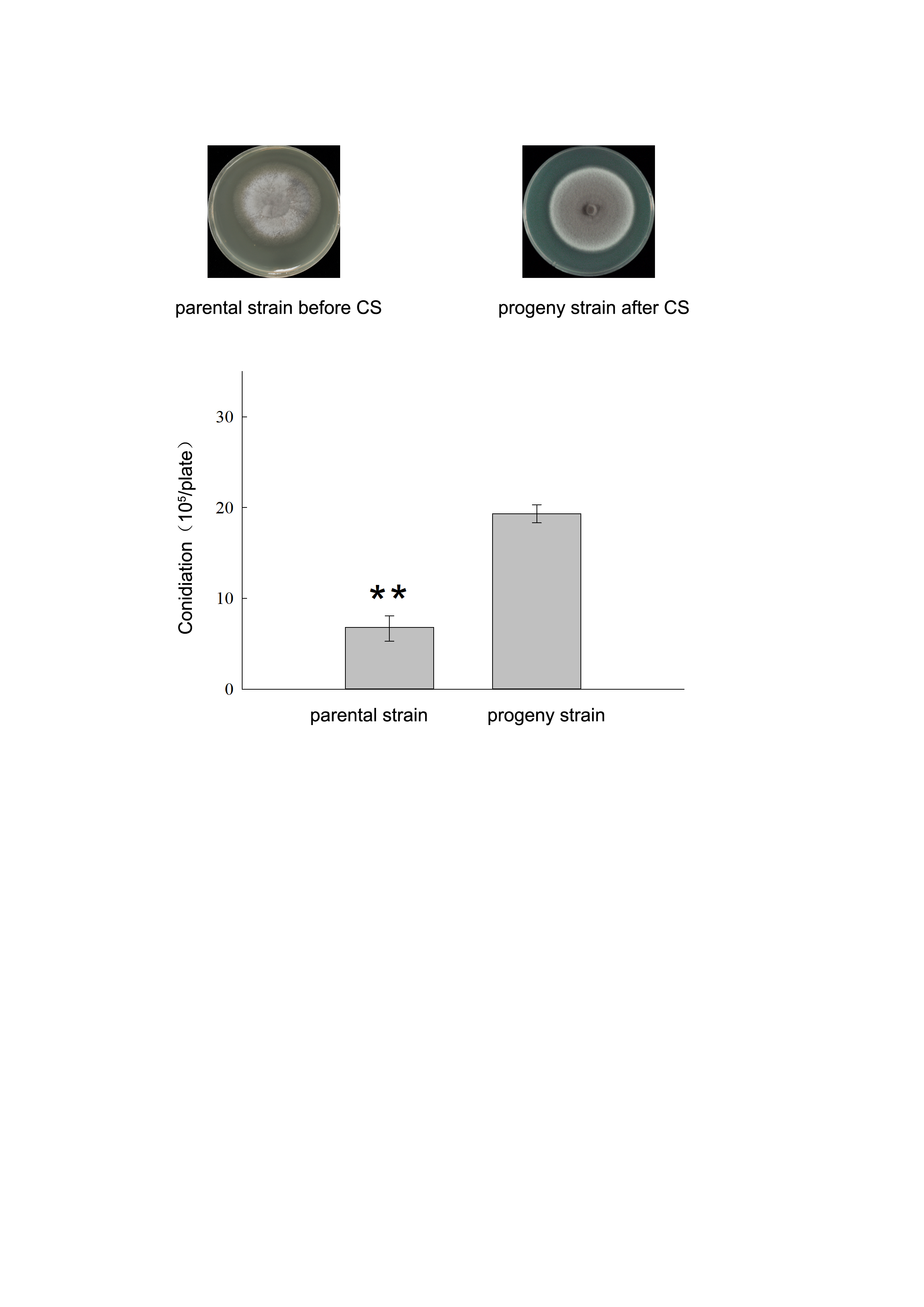

Supplement: S1 Fig — An Magnaporthe oryzae Guy-11 strain degenerated during repeat subculturing were treated by CS. After CS treatment, the colonial morphology and conidiation of the strains were improved were improved obviously. (TIF) [file pone.0224635.s001.tif]
